# The London County Council and Hospital Assessment

**Published:** 1891-03-14

**Authors:** 


### PASSING TOPICS.

### THE LONDON COUNTY COUNCIL AND HOSPITAL ASSESSMENT.

The action of the London County Council in proposing a general increase of assessment, whether justified or no in regard to property at large, will prove, if pushed to its logical extreme, a serious and sorry matter for our hospitals. There are cases in which it is proposed to double or treble assessments already sufficiently heavy, and thus to accentuate still further the grievous injustice which some of our most useful institutions suffer by an impost, inequitable in principle and rendered still more galling by an incidence which, if not the result of actual caprice, is at best altogether unintelligible. Forbid it that any word of ours should appear to invite the taxation of a hospital now only nominally assessed. There are institutions which can be thus classed, and we congratulate them, but why, it may be reasonably demanded by the rest, should justice, equity, generosity, or, if you will, prodigality, flourish on one side of a parish boundary, while on the other we find nothing but a hard Gradgrind policy which declines to lighten by a single grain the heavy array of insoluble facts which go to make up the full measure of our hospitals' poverty?

Even within the limits of a single parish, there are inequalities of rating to be found, which are nothing less than startling, and unless the County Council are prepared to deal equally all round, either by the levelling up or the reverse process, they will find some difficulty about satisfying the love of justice which we would fain hope survives in our midst, and can upon occasions make itself both heard and felt. We believe there is a Charity Rate Exemption Society hidden away somewhere, though strangely enough it makes no sign at this juncture which, we should have thought, ought to galvanise into action any organisation of the kind not actually moribund.

Poor hospitals! Whatever other virtues are denied to them, they must at least be credited with meekness and submission, even though the meekness is born of that poorness of spirit which is akin to despair. Were they railway companies with enormous resources and a plethora of powers and privileges, they would anathematise the Government with vehemence, and quietly transfer the burden of the taxation to the shoulders of their passengers—a superlatively wise policy, which enlarges the area of discontent, and rallies many who otherwise would be neutral. Hospitals have no such ready means of deliverance. It would be as impracticable to levy a tax upon their patients as upon their subscribers. What cannot be cured must be endured, and if endured with a good grace, why so much the better, as it seems, and the easy indifference with which St. Mary's, for instance, has accepted a new assessment multiplying the old by six, is remarkable. Of course, if assessed at all, £1,500 is not an outrageous figure for a hospital of 285 beds, but how is the system to be justified, which permits of virtual freedom for many years, only to be followed by so rude a rivetting of bonds? The bogey man in the song is not more non-existent than a system:—obviously "there ain't none." Every parish is a law to itself. We once knew an instance in which an institution lived a few years unassessed, because the overseers of the time were friendly people, and it was said the institution was not a bad customer. Ah! those were golden days; but times and overseers have changed and Nemesis has overtaken the place long ago.

Upon consideration we are almost persuaded that the very best thing which could come about in this connection would be for the Council to persevere in a blind, impartial, up to the hilt assessment of every hospital in the metropolis, because we think such action would compel attention and a remedy. Have the County Council the courage to make the experiment? Agricola.

